# Cancer du sein en Mauritanie: série rétrospective de 200 cas au Centre National d’Oncologie de Nouakchott

**DOI:** 10.11604/pamj.2025.52.36.45258

**Published:** 2025-09-24

**Authors:** Ahmed El Heiba Mamina, Fatimetou Mohamed El Hassen, Jemila Bouka, El Hadj Menny, Cheikh Sid' Ahmed Tolba

**Affiliations:** 1 Faculté de Médecine, de Pharmacie et d’Odonto-Stomatologie, Nouakchott, Mauritanie,; 2 Service d’Anatomie et de Cytologie Pathologique, Centre National d’Oncologie (CNO), Nouakchott, Mauritanie,; 3 Hôpital Militaire de Nouakchott, Mauritanie

**Keywords:** Cancer du sein, Mauritanie, statut des récepteurs hormonaux

## Aux éditeurs du Pan African Medical Journal

Le cancer du sein demeure le cancer le plus fréquent et la première cause de mortalité par cancer chez la femme dans le monde [1]. En Afrique subsaharienne, il représente un véritable défi de santé publique en raison du diagnostic tardif, du manque d'infrastructures et de l'accès limité aux traitements adaptés [2]. En Mauritanie, il constitue également le cancer féminin le plus fréquent, représentant 30,9% des cancers féminins et 16,56% de l'ensemble des cancers recensés au niveau national [3]. Malgré l’impact majeur du cancer du sein en Mauritanie, les données cliniques et pathologiques restent rares. Cette étude rétrospective portant sur 200 cas suivis au Centre National d’Oncologie sur deux années comble ce vide en analysant les caractéristiques épidémiologiques, cliniques et anatomopathologiques, afin d’éclairer les politiques de santé publique et d’optimiser la prise en charge.

Au Centre National d’Oncologie (CNO) de Nouakchott, principal centre de référence national, 429 cas de cancer du sein ont été recensés entre 2020 et 2021, représentant 15,71% de l'ensemble des cancers diagnostiqués. La présente lettre expose les principaux résultats d’une étude rétrospective analytique menée sur 200 patientes disposant de dossiers cliniques et anatomo-pathologiques exploitables. Les données issues de cette série montrent des similitudes notables avec les tendances rapportées dans d’autres pays d'Afrique subsaharienne [2]. Parmi les 200 patientes incluses, 98,5% étaient de sexe féminin. L'âge moyen était de 50,46 ans, avec 32% des cas survenant entre 45 et 55 ans. Ces résultats sont comparables à ceux rapportés au Burkina Faso (âge moyen: 50 ans) [4] et au Cameroun (46,8 ± 15,87 ans) [5], mais restent inférieurs à ceux observés dans les pays occidentaux [6].

Environ un tiers des patientes ont consulté entre 3 et 6 mois après l'apparition des premiers signes, un délai prolongé classiquement observé dans de nombreux pays à ressources limitées où les barrières à l'accès aux soins et le manque de sensibilisation restent marqués [7]. Sur le plan histologique, 85% des cas étaient des carcinomes canalaires infiltrants de type non spécifié (NST), en accord avec les données de la littérature régionale [7,8]. Le profil immunohistochimique était disponible pour 102 patientes (51%). Parmi elles, 52,94% étaient ER-négatives, 58,82% PR-négatives et 20,6% HER2-positives (dont 3 cas évalués à 2+ en IHC, avec deux amplifications confirmées par FISH). Le Ki-67 a été évalué chez 55 patientes. Globalement, 35,3% des cancers étaient de phénotype triple négatif, posant un défi thérapeutique majeur. Cette forte prévalence du phénotype triple négatif (TNBC) est conforme aux données africaines, où ce sous-type est plus fréquent que dans les pays occidentaux [8].

Bien que 70,5% des patientes aient allaité et que 31,5% soient multipares, l'effet protecteur généralement associé à ces facteurs semble limité dans cette cohorte, probablement en lien avec la prédominance des tumeurs RH-négatives. Ma *et al*. ont déjà rapporté que cet effet protecteur n´était significatif que dans les cancers RH- positifs [9]. L'absence de données précises sur la durée de l'allaitement limite cependant l'interprétation. Cette étude met en évidence deux faiblesses majeures dans la prise en charge du cancer du sein en Mauritanie: d'une part, un accès tardif au diagnostic, avec un tiers des patientes consultant après trois mois ou plus; d'autre part, une offre d´immunohistochimie encore partielle, introduite de manière stable seulement en 2024. La fréquence élevée des phénotypes triple négatifs observée dans cette cohorte pourrait être liée non seulement à des facteurs biologiques propres à la population, mais aussi à des biais techniques, notamment un risque de faux négatifs associé à des conditions préanalytiques sous-optimales. L'immunomarquage reste actuellement limité aux récepteurs hormonaux (ER, PR), à HER2 et à Ki-67, sans possibilité de recours à d'autres marqueurs immunohistochimiques, pourtant essentiels en cas d'infiltration suspectée sur carcinome in situ. Par ailleurs, les techniques complémentaires telles que le FISH ne sont pas réalisées localement.

Malgré les limites inhérentes à la nature rétrospective de l'étude et aux données incomplètes concernant certains biomarqueurs, ce travail constitue l'une des premières synthèses nationales sur le cancer du sein en Mauritanie. Il pourrait ainsi servir de base à l'élaboration de futures stratégies de dépistage, d'orientation diagnostique et de recherche clinique.

Cette série rétrospective met en lumière les spécificités du cancer du sein en Mauritanie et les défis liés au diagnostic et à l'accès aux biomarqueurs. Elle constitue une base utile pour renforcer les stratégies locales de détection précoce, améliorer l'offre diagnostique, et stimuler la recherche clinique adaptée aux réalités des pays à ressources limitées.

## Remerciements

Les auteurs remercient les équipes médicales et administratives du Centre National d'Oncologie pour leur soutien.

## References

[1] Sung H, Ferlay J, Siegel RL, Laversanne M, Soerjomataram I, Jemal A (2021). Global Cancer Statistics 2020: GLOBOCAN Estimates of Incidence and Mortality Worldwide for 36 Cancers in 185 Countries. CA: A Cancer Journal for Clinicians.

[2] Sharma R, Aashima, Nanda M, Fronterre C, Sewagudde P, Ssentongo AE (2022). Mapping Cancer in Africa: A Comprehensive and Comparable Characterization of 34 Cancer Types Using Estimates From GLOBOCAN 2020. Front Public Health.

[3] Brahim SM, Hamed CT, Zein E, Salame M, Veten F, Zein MV (2021). Epidemiological and Clinicopathological Features of Breast Cancer in Mauritania.

[4] Sano D, Lankoande J, Dao B, Cisse R, Traore SS, Soudre RB (1997). Le cancer du sein, problèmes diagnostiques et thérapeutiques au CHU de Ouagadougou. Médecine d´Afrique Noire.

[5] Engbang JP, Essome H, Koh VM, Simo G, Essam JD, Mouelle AS (2015). Cancer du sein au Cameroun, profil histo-épidémiologique : à propos de 3044 cas [Breast cancer in Cameroon, histo-epidemiological profile: about 3044 cases]. Pan Afr Med J.

[6] Dafni U, Tsourti Z, Alatsathianos I (2019). Breast Cancer Statistics in the European Union: Incidence and Survival across European Countries. Breast Care (Basel).

[7] Diakité N (2011). Cancer du sein: aspects cliniques et thérapeutiques dans le Service de Chirurgie A du CHU du Point G. ADHL.

[8] Gutnik L, Olopade OI, Newman LA, Fayanju OM (2022). Breast cancer among African American and sub-Saharan African women: a tale of global inequities. Cancer Causes Control.

[9] Ma H, Bernstein L, Pike MC, Ursin G (2006). Reproductive factors and breast cancer risk according to joint estrogen and progesterone receptor status: a meta-analysis of epidemiological studies. Breast Cancer Res.

